# An Essay on the Chemical History and Medical Treatment of Calculous Disorders

**Published:** 1818-05

**Authors:** 


*An Essay on the Chemical History and Medical Treatment of Calculous Disorders.* By ALEXANDER MARCET, M. D. F. R. S. Physician to Guy's Hospital, &c. &c. pp. 181. London, 1817.

THE knowledge of which we are already possessed relating to the composition of urinary concretions is considerable; but there is, nevertheless, much that remains unknown, with regard to the practical, and consequently the most useful, application of our information.

The author of the present treatise states, in the Introduction, that his object is to "describe and illustrate, by means of accurate engravings, the characters by which the different calculi may be distinguished; to indicate the easiest analytical methods by which their chemical nature may be ascertained; and to point out the modes of medical treatment which afford the best prospect of success."

The author takes some pains to assure the profession, that the present Essay must not be regarded as any encroachment upon the province of surgery; a care which, in our opinion, might have been spared, as the Doctor appears to have trodden, with light and cautious step, any ground that might particularly concern the practical surgeon. In matter, however, the work is not uninteresting to the chemical pathologist, and as to style it is pleasantly written.

The Essay is divided into eight chapters, which we shall now proceed to notice in their respective order.

CHAP. 1. *On the Seat of Calculi, and on the Symptoms produced.*

Under this head nothing particularly new is presented to our notice. We find that calculous concretions are found in the kidney, ureter, urinary bladder, or urethra. The only situations in which calculous matter is found in the kidney are stated to be the infundibuli or pelvis, in which position the author agrees with every writer we know of upon the subject.

In describing the effects produced by the presence of large calculi in the kidney, he refers to a preparation represented on the second plate, and from this concludes, that "when such complete alteration of structure takes place, the secretion of urine must, *of course*, be carried on by the other kidney." Upon this point, however, we cannot agree with the author, for we have seen instances, and been present at dissections which, although not necessary to detail, afforded proof positive that it is extremely difficult, if not totally impossible, to define the degree of apparent change in the glandular structure of the kidney, which is incompatible with the secretion of urine.

Calculi are also occasionally found in the ureters, where, when they are small, they pass with comparative ease; when large, they create irritation. Where, from inflammation, the structure of the ureter has been changed, the Doctor has found its inner surface partially covered with a crust of calculous matter.

The most frequent seat of calculus is the urinary bladder, in which the stone is found either loose or encysted. An interesting history of an old gentleman, who for many years suffered from irritation in the urinary passages, is given, in which there was no diagnostic symptom of stone, although the patient sunk at last from increase of irritation, and died. On examination, an encysted stone was found weighing 3083 grains, and composed of two masses of lithic acid, united by an intervening layer of crystallized triple phosphat.

The author also acquaints us, that calculi are occasionally arrested in their passage through the urethra, and that, under these circumstances, they may and do require to be removed by an operation; and that, now and then, these small calculi are even found within the cavities of a diseased prostate gland.

The symptoms are next considered.

In the kidney, the general indications of calculus are, continued pains in the region of the loins, purulent urine, hæmorrhage, &c. The symptoms of a calculus passing by the ureter we shall describe, as affording a favourable specimen of our author's style.

"It is probably during the passage of a calculus from the kidnies to the bladder, rather than during its formation, that the greatest pain is experienced. In the latter case, the pain felt in the lumbar region is rather of the obtuse kind, whilst during the descent of a calculus into the bladder, it is sometimes very pungent, and is very apt to shoot downwards, in the direction of the ureters. In either case, the disease is frequently attended with a drawing up of the testicle, and a sense of numbness in the thigh on the affected side. The urine is generally of a deep red colour; it is voided frequently, and in small quantities at a time, and it often deposits a brick-coloured sediment."

The symptoms enumerated, as indicating the presence of stone in the bladder, are uneasy sensations in the glans penis, frequent desire to void urine, and sudden stoppage of its passage. The symptoms excited by an encysted calculus are variously modified.

The author endeavours to establish a diagnostic for calculi existing in the prostate gland, but his account has nothing in it which to us appears satisfactory or interesting.

Chap. 2. *Of the different Prevalence of Urinary Calculi, in various Districts and Hospitals; and of the comparative Frequency of the Disease in different Countries.*

Under this head, the author takes a comparative, and as we think, rather a dull, survey of the comparative frequency of stone, as occuring in the various London hospitals, the Norwich Infirmary, and other establishments of a similar description, abroad as well as at home; and the result of his inquiry is, " a suspicion that the tendency to form urinary calculi must arise from some general causes, independent of any of the peculiarities of food or beverage to which they have been usually ascribed." He also conceives, that, from the infrequency of the disease in tropical latitudes, there is probably some essential connection between the functions of the skin and those of the kidnies.

Chap. 3. *Of the different Species of Urinary Calculi— of their external Characters — of their chemical Nature and Classification.*

The figure, and general external appearance of calculi, differ according to the circumstances of their situation and number, whether they are found in the kidney, bladder, or elsewhere. Where the colour is brownish, or like mahogany, the calculus generally consists of lithic acid. The white, or greyish white, indicates the earthy phosphats; and when the calculus is of a dark brown or black colour, it consists of oxalate of lime. The uneven crystalline surface denotes the presence of the triple phosphats.

The author states, that the specific gravity of calculi varies between 1200 and 1900; water being 1000. As to structure, calculi differ considerably, the nucleus generally indicating its having originated in the kidney, although sometimes found to be a foreign body accidentally introduced into the bladder.

The following is the arrangement adopted by the author, in considering the composition of calculi :—1. The *lithic* calculus. 2. The *bone-earth* or phosphat of lime calculus. 3. The *ammoniaco-magnesian phosphat* calculus. 4. The *fusible* calculus, a mixture of the two former. 5. The *mulberry* calculus, oxalat of lime. 6. The *cystic* calculus, or cystic oxyd. 7. The *alternating* calculus, where the substances are arranged in layers. 8. The *compound* calculus, the ingredients not separable without chemical analysis. 9. *Prostate* calculi.

The author next proceeds to the more particular consideration of the properties of each kind of calculus; but the detail, although it evinces much information, is calculated to afford little interest, except to the professed chemist. We shall, therefore, upon this head, refer the reader to the work itself. Several cases are related; and with additional observations, Dr. Marcet finishes this division of his subject.

### Chap. 4. *An Account of two Calculi, which cannot be referred to any of the Species hitherto described.*

The first specimen of this kind was received by the author from Dr. Babington, a physician whose amiable manners, no less than his professional talents, render him deserving of every respect. The author details the result of a series of analytic experiments upon this calculus, which, however, can only be understood by reference to the work itself. The conclusion formed is, "that it is a substance *sui generis,* and will probably be found entitled to be considered as an oxyd, though it is certainly much less soluble in acids than the cystic oxyd." For this the Doctor proposes the name of *Xanthic Oxyd,* from ξανθος, *yellow;* from its allusion to a striking property (that of forming a lemon-coloured compound when acted upon by nitric acid).

The other non-descript calculus was also submitted to experiment, and leads the author to conclude, that "all its properties correspond exactly to those of fibrine, and, therefore, if the occurrence of similar concretions should render it necessary to give them a name, they might, without impropriety, be called *fibrinous calculi.* The outline of the case, in which this last described calculus was passed, is also detailed, but has nothing in it peculiar. The manner, however, in which the properties of these two newly ascertained species of calculi were determined by experiment, prove the author to be an excellent chemist, desirous of applying his extensive information to a most useful purpose.

CHAP. 5. *On the comparative Frequency of the different Species of Urinary Calculi.*

The determination of this question, the Doctor observes, is a point of no small difficulty. The work of extensive and comprehensive analysis is one of a tedious, and, in the opinion of some individuals possessing collections, rather objectionable nature. These are difficulties which it is proper every writer should mention where they occur, and equally proper for every reader to take into the account; for, as such obstacles require zeal and perseverance in him who determines to surmount them, they enable the diligent inquirer to lay the fairest claim upon the approbation and applause of the thinking part of mankind, for whatever success may crown his pursuits.

The author brings forward an exceedingly interesting Table, exhibiting the composition of 181 calculi examined in the Norwich collection. In the operations for the extraction of these calculi, 25 are stated to have been fatal cases, a proportion of 1 in 7½.

The lithic calculi were 66 in number. Those composed of the phosphats 4, of fusible calculi 49, mulberry calculi 41, of variously disposed mixtures 21; total 181.

The inferences drawn from the above-mentioned Table of Cases are interesting; Dr. Marcet concludes them with the following valuable observation:

"The result is the more curious, as it seems to shew, that it is not so much the mechanical irritation of the stone, as the particular diathesis of the urinary secretions which influences the event of the operation.

In the calculi, in the collection at Guy's hospital, 22 were lithic calculi, 3 phosphat of lime, 2 triple phosphat, 24 fusible calculi, 22 mulberry, 13 compound, 1 cystic oxyd; total 87.

The proportion of lithic calculi is observed to be much smaller in this than the Norwich collection, which tends to shew, that the calcareous nature of the water in the eastern counties of England, to which the greater prevalence of the stone in these districts has been generally ascribed, is probably in nowise connected with this disease, since the earthy species of calculus is comparatively much more frequent in London.

( To be concluded in our next. )

